# Diagnostic Accuracy of Dried Plasma Spot Specimens for HIV-1 Viral Load Testing: A Systematic Review and Meta-analysis

**DOI:** 10.1097/QAI.0000000000002855

**Published:** 2021-11-03

**Authors:** Youyi Fong, Jessica Markby, Mauro Andreotti, Ingrid Beck, Thomas Bourlet, Don Brambilla, Lisa Frenkel, Rosalia Lira, Julie A. E. Nelson, Georgios Pollakis, Sandrine Reigadas, Douglas Richman, Souleymane Sawadogo, Laura Waters, Chunfu Yang, Clement Zeh, Meg Doherty, Lara Vojnov

**Affiliations:** a Fred Hutchinson Cancer Research Center, Seattle, WA;; b Clinton Health Access Initiative, Boston, MA;; c National Center for Global Health, Istituto Superiore di Sanita, Rome, Italy;; d Center for Global Infectious Disease Research, Seattle Children's Research Institute, Seattle, WA;; e Laboratory of Virology, University Hospital of Saint-Etienne, France;; f RTI International, Rockville, MD;; g Departments of Pediatrics and Laboratory Medicine, University of Washington, Seattle, WA;; h Unidad de Investigacion Medica en Enfermedades Infecciosas y Parasitarias, UMAE Hospital de Pediatria, CMN Siglo XXI, Instituto Mexicano del Seguro Social (IMSS), Mexico City, Mexico;; i Department of Microbiology and Immunology, School of Medicine, University of North Carolina at Chapel Hill, Chapel Hill, NC;; j Department of Human Retrovirology, University of Amsterdam, Amsterdam, the Netherlands;; k Laboratory of Virology, University Hospital of Bordeaux 33076, Bordeaux, France;; l University of California San Diego, La Jolla, CA;; m Division of Global HIV/AIDS, US Centers for Disease Control and Prevention, Windhoek, Namibia;; n St. Stephens AIDS Trust, Chelsea & Westminster Hospital, London, United Kingdom;; o International Laboratory Branch, Division of Global HIV/AIDS and TB, Center for Global Health, Centers for Disease Control and Prevention, Atlanta, GA;; p Division of HIV/AIDS Prevention, US Centers for Disease Control and Prevention, Kisumu, Kenya; and; q World Health Organization, Geneva, Switzerland.

**Keywords:** viral load, dried plasma spot, systematic review

## Abstract

**Background:**

Dried plasma spot specimens may be a viable alternative to traditional liquid plasma in field settings, but the diagnostic accuracy is not well understood.

**Methods:**

Standard databases (PubMed and Medline), conferences, and gray literature were searched until January 2019. The quality of evidence was evaluated using the Standards for Reporting Studies of Diagnostic Accuracy and Quality Assessment of Diagnostic Accuracy Studies-2 criteria. We used univariate and bivariate random effects models to determine misclassification, sensitivity, and specificity across multiple thresholds, overall and for each viral load technology, and to account for between-study variation.

**Results:**

We identified 23 studies for inclusion in the systematic review that compared the diagnostic accuracy of dried plasma spots with that of plasma. Primary data from 16 of the 23 studies were shared and included in the meta-analysis, representing 18 countries, totaling 1847 paired dried plasma spot:plasma data points. The mean bias of dried plasma spot specimens compared with that of plasma was 0.28 log_10_ copies/mL, whereas the difference in median viral load was 2.25 log_10_ copies/mL. More dried plasma spot values were undetectable compared with plasma values (43.6% vs. 29.8%). Analyzing all technologies together, the sensitivity and specificity of dried plasma spot specimens were >92% across all treatment failure thresholds compared and total misclassification <5.4% across all treatment failure thresholds compared. Some technologies had lower sensitivity or specificity; however, the results were typically consistent across treatment failure thresholds.

**Discussion:**

Overall, dried plasma spot specimens performed relatively well compared with plasma with sensitivity and specificity values greater than 90% and misclassification rates less than 10% across all treatment failure thresholds reviewed.

## Abstract 2

Supplemental Digital Content is Available in the Text.

## INTRODUCTION

Of the nearly 38 million people living with HIV, approximately 24.5 million had access to antiretroviral therapy in 2019.^1^ Monitoring treatment is critical to ensure people on antiretroviral therapy are on the most effective regimen. Furthermore, achieving viral suppression reduces the risk of onward transmission.^2^ Global targets now exist to evaluate the effectiveness of identifying and treating people living with HIV. The last 90 of UNAIDS′ 90-90-90 targets measures the proportion of people on antiretroviral therapy who are virally suppressed.^3^ Increasing access to viral load testing is essential to support high-quality individual treatment monitoring and to understand individual and overall population suppression rates to minimize transmissions.

The 2016 World Health Organization (WHO) consolidated guidelines on the use of antiretroviral drugs for treating and preventing HIV infection recommend viral load as the preferred monitoring approach to diagnose and confirm treatment failure, with plasma specimens as the preferred specimen type.^4^ Although viral load testing has scaled up considerably in low-income and middle-income countries,^5^ several challenges remain. In particular, the use of traditional liquid plasma can be difficult for some countries or settings because of strict specimen storage and transport times and temperatures. Most manufacturers of currently approved viral load assays require plasma separation from whole blood within 24 hours of specimen collection.^6^ These requirements, therefore, limit the breadth and scope of viral load testing programs. An analysis across 4 sub-Saharan African countries found that approximately only 44% of health care facilities and 52% of people on antiretroviral therapy can access viral load testing using plasma specimens under those conditions. Alternative specimen types and technologies will be critical to support expansion of viral load testing to all in need as national infrastructural projects further develop to allow for improved and expedited transport.

Dried plasma spot cards are an alternative specimen type that requires the application of liquid plasma to a filter paper card. They are similar to dried blood spot cards and specimens, except that plasma rather than whole blood is applied directly to the card. Together with dried blood spot specimens, dried plasma spot specimens may be able to support wider decentralization and access to viral load testing; however, they typically require a centrifuge to separate the plasma from whole blood before application. Dried plasma spot specimens do not require cold chain, can be stored for longer periods of time once prepared, and are safer to transport because they are generally no longer infectious. In addition, they can be prepared by lower cadres of health care facility staff, similar to dried blood spot specimens and point-of-care technologies, further allowing decentralization and task-shifting.^7–10^

Several diagnostic accuracy studies have been published highlighting the performance of dried plasma spot specimens compared with that of traditional liquid plasma for HIV-1 viral load testing in people living with HIV.^11–33^ Given the significant interest and effort in scaling up viral load testing in resource-limited settings, it was timely to collate and summarize the findings through a systematic review and meta-analysis.

## METHODS

### Search Strategy

A systematic review and meta-analysis was performed according to the Preferred Reporting Items for Systematic Reviews and Meta-Analyses following a predefined study protocol^34^ (see Table 1, Supplemental Digital Content, http://links.lww.com/QAI/B756). A search was conducted in January 2019 using PubMed and Medline databases to identify peer-reviewed original research with appropriate data for this systematic review and meta-analysis (search terms given in Supplemental Digital Content 1, http://links.lww.com/QAI/B755). Conference abstracts from the Conference on Retroviruses and Opportunistic Infections, International Conference on AIDS and sexually transmitted infections in Africa, International AIDS Society, and AIDS Conferences and extensive bibliography and gray literature were screened for possible inclusion. No restrictions on publication year, publication status, or language were used.

For inclusion, studies must have compared viral load values using dried plasma spot specimens with the reference standard of liquid plasma specimens and measured by 1 or more of the following 7 commonly used technologies—Abbott RealTime HIV-1 on the m2000 platform (Abbott Molecular Inc, Abbott Park, IL), Generic HIV Charge Virale (Biocentric, Bandol, France), bioMérieux NucliSENS EasyQ HIV-1 v2.0 (bioMérieux, Craponne, France), Cavidi ExaVir Load (Cavidi, Uppsala, Sweden), Hologic Aptima (Hologic, Marlborough, MA), Roche Amplicor HIV-1 Monitor Test, v1.5 or COBAS AmpliPrep/COBAS TaqMan HIV-1 Test, v2.0 (Roche Molecular Systems Inc, Basel, Switzerland), and/or Siemens VERSANT HIV-1 RNA 1.0 assay (kPCR) (Siemens Healthcare Diagnostics, Munich, Germany). No studies were found that used the Siemens VERSANT HIV-1 RNA 1.0 assay. Studies were not included if the index assay used was an in-house developed assay that lacks any international regulatory approval and/or cannot be procured traditionally by other countries or laboratories.

### Study Selection and Systematic Review

Two reviewers independently screened all titles and abstracts for inclusion and reviewed all potentially relevant studies in full. Studies were included if they evaluated the accuracy of dried plasma spot specimens compared with that of traditional liquid plasma, were pertaining to viral load testing, and were performed using plasma prepared from blood sample of HIV-positive patient. Studies were excluded if they used spiked or pooled blood specimens or panels, they compared dried plasma spot specimens with plasma using a different assay, they performed a qualitative analysis of dried plasma spot specimens, or the comparator was a sample type other than liquid plasma.

Data were extracted and summarized for all included studies, outlining the study design, methods, and principle components of each study (eg, sample size, viral load assay used, and storage and transport conditions of specimens). Study characteristics were extracted from each manuscript or through author contact, when necessary. The primary outcome assessed was accuracy of the dried plasma spot specimens compared with that of plasma. Forest plots of the log bias and r-squared variables were developed to analyze the between-study heterogeneity of diagnostic performance.

**FIGURE 1.**
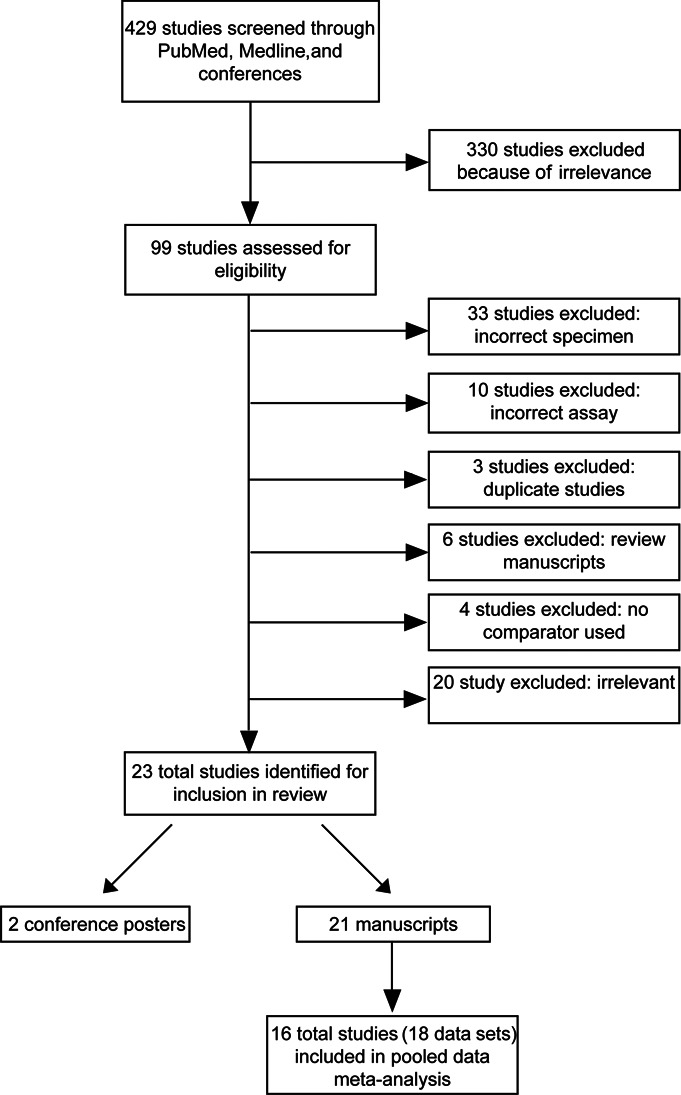
PRISMA flow chart of titles screened and studies included.

Twenty-three studies were identified through the search strategy (Fig. 1). We contacted the corresponding authors of all studies that met the inclusion criteria at least twice to explain the analysis plan and request primary data. For the meta-analysis, a total of 16 studies provided 18 data sets across 6 technologies resulting in a total of 1847 paired dried plasma spot and plasma viral load results. Data from the remaining 7 studies were not included in the meta-analysis because the study authors did not respond to the request to share.

### Quality Assessment

The Standards for Reporting Studies of Diagnostic Accuracy criteria and Quality Assessment of Diagnostic Accuracy Studies-2 were followed and each study graded for quality.^35,36^

### Statistical Analyses—Meta-Analysis

Study variables reviewed for each study included study sample size, viral load mean and median, proportion of patient specimens within specific viral load ranges, and sensitivity and specificity. Sensitivity and specificity were previously defined.^9^ In brief, sensitivity was calculated as the proportion of dried plasma spot specimens correctly identified as failing or above the defined virological failure threshold compared with that of plasma. Specificity was calculated as the proportion of dried plasma spot specimens correctly identified as not failing or below the virological failure threshold compared with that of plasma. Primary data were then pooled to analyze the performance of dried plasma spot specimens for each technology. Viral load values were log-transformed because of the nonnormal distribution of the data. Because longitudinal data on dried plasma spot specimen performance were not available, cross-sectional comparisons were performed. In addition, lower treatment failure thresholds for viral load using dried plasma spot specimens were assessed including detectable (defined as any detectable result indicating treatment failure), 200, 400, 500, 600, 800, and 1000 copies/mL. Performance of dried plasma spot specimens was compared with that of plasma across each treatment failure threshold with measurements of true positives, true negatives, false positives, and false negatives calculated for each technology to create estimates of diagnostic accuracy of dried plasma spot specimens overall and for each platform across all studies. Using these treatment failure thresholds, the sensitivity, specificity, upward and downward misclassification rates, and positive and negative predictive values were also calculated. Upward misclassification was defined as the number of dried blood spot specimens incorrectly identified as above the tested treatment failure threshold divided by the total number of matched plasma specimens with viral load results less than 1000 copies/mL. Downward misclassification was defined as the number of dried blood spot specimens incorrectly identified as below the tested treatment failure threshold divided by the total number of matched plasma specimens with viral load results more than 1000 copies/mL.

Random effects models were used to estimate the summary measures for accuracy accounting for between-study variation. For sensitivity and specificity values and corresponding 95% confidence intervals (CIs), bivariate random effects models designed to estimate summary sensitivity and specificity were used to simultaneously determine the estimates, accounting for the covariance of sensitivity and specificity and study-specific heterogeneity.^37^ To obtain estimates of misclassification, univariate random effects models were used to obtain the point estimates and corresponding 95% CIs.^38–40^ Graphic representations were completed in GraphPad Prism (La Jolla, CA), and analyses were completed in R version 3.4.3 (The R Foundation).

### Protocol

The prepared protocol was reviewed by the World Health Organization and approved by Chesapeake Institutional Research Review Board (Columbia, MD; www.chesapeakeirb.com).^9^

## RESULTS

### Systematic Review

After screening 429 peer-reviewed publications and conference abstracts, we identified 23 studies that met our inclusion criteria and were published between 1997 and 2017 (Fig. 1 and Table 1). The excluded studies were those that used incorrect specimen types (33) or incorrect assays (10), duplicates (3), review manuscripts (6), or had no comparator included (4).

**TABLE 1.** Study Characteristics and Dried Plasma Spot Specimen Preparation Protocol for Each Included Study

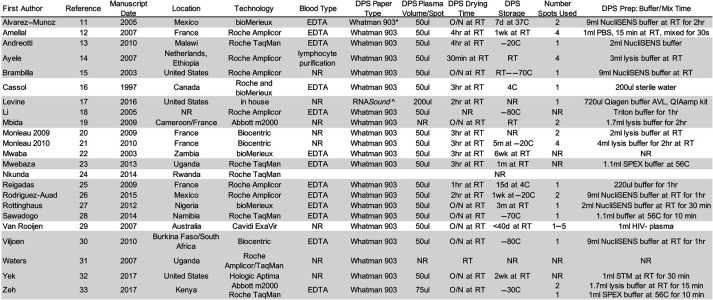

Gray shading indicates the studies that shared primary data for the meta-analysis.

NR, not reported; O/N, overnight; RT, room temperature; SPEX, specimen preextraction reagent (proprietary); STM, specimen transfer medium (proprietary)

*Sigma-Aldrich, St. Louis, Missiouri.

†FortiusBio, San Diego, California.

Studies were reasonably distributed geographically with 47.8% of studies including study participants from Africa,^13,14,19,22–24,27,28,30,31,33^ 26.1% from Europe,^12,14,19–21,25^ and 26.1% from the United States/Canada/Mexico.^11,15–17,26,32^ Most studies used the Roche Amplicor HIV-1 or COBAS TaqMan HIV-1 technologies (56.5%; 13),^12–16,18,23–26,28,31,33^ and 34.8% (8) used the now discontinued Roche Amplicor HIV-1 technology.^12,14–16,18,25,26,31^ Approximately 26.1% (6) used the Roche COBAS TaqMan HIV-1 technology,^13,23,24,28,31,33^ 13.0% (3) used the Biocentric Generic HIV Charge Virale technology,^20,21,30^ and 17.4% (4) used the bioMerieux NucliSENS EasyQ HIV-1 technology.^11,16,22,27^ Two studies used the Abbott RealTime HIV-1 technology,^19,32^ whereas 1 study each used the Cavidi ExaVir Load^29^ and Hologic Aptima^32^ technologies.

### Quality of Studies

The quality assessment found some risk of bias in patient selection, reference standard, and index test (Supplemental Digital Content Fig. 1, http://links.lww.com/QAI/B754). In most studies, it was unclear regarding blinding and the timing of testing, whereas few stated how specimens were selected— and inclusion and exclusion criteria were often lacking. Furthermore, study design and patient/specimen demographics were rarely stated or presented. In addition, most studies were conducted before 2011 (15: 65.2%). There was, however, applicability in patient selection, index test, and reference standard in most studies.

### Systematic Review Analysis

**FIGURE 2.**
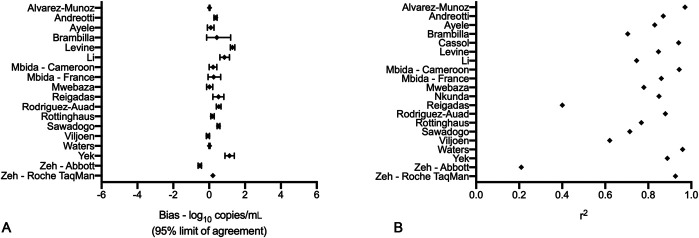
Forest plots of log bias (A) and linear regression (r2) (B) of all studies with included metrics.

The median study sample size was 47 specimens. The primary metrics conducted and included in studies were the linear regression (r^2^) and log bias analyses (Fig. 2 and Table 2); however, no metric was consistently presented across all studies. Nearly all studies included only quantitative analytics and were published before the WHO recommendations; therefore, none presented data regarding a treatment failure threshold as is currently recommended by the WHO and practiced across most low-income and middle-income countries.

**TABLE 2.** Analytical and Clinical Metrics for Each study

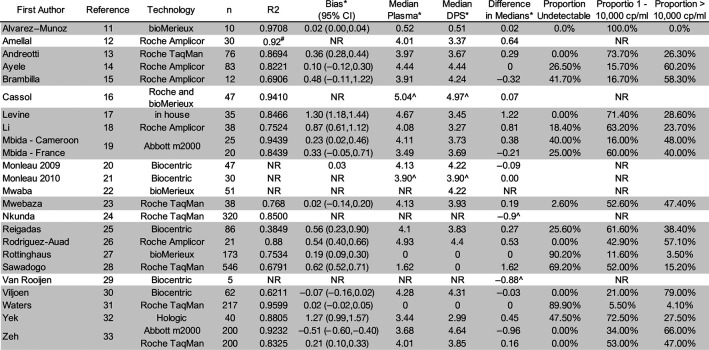

Those in gray shading provided primary data for the meta-analysis.

*log_10_ copies/mL.

#Rho.

^Mean.

### Meta-Analysis

**FIGURE 3.**
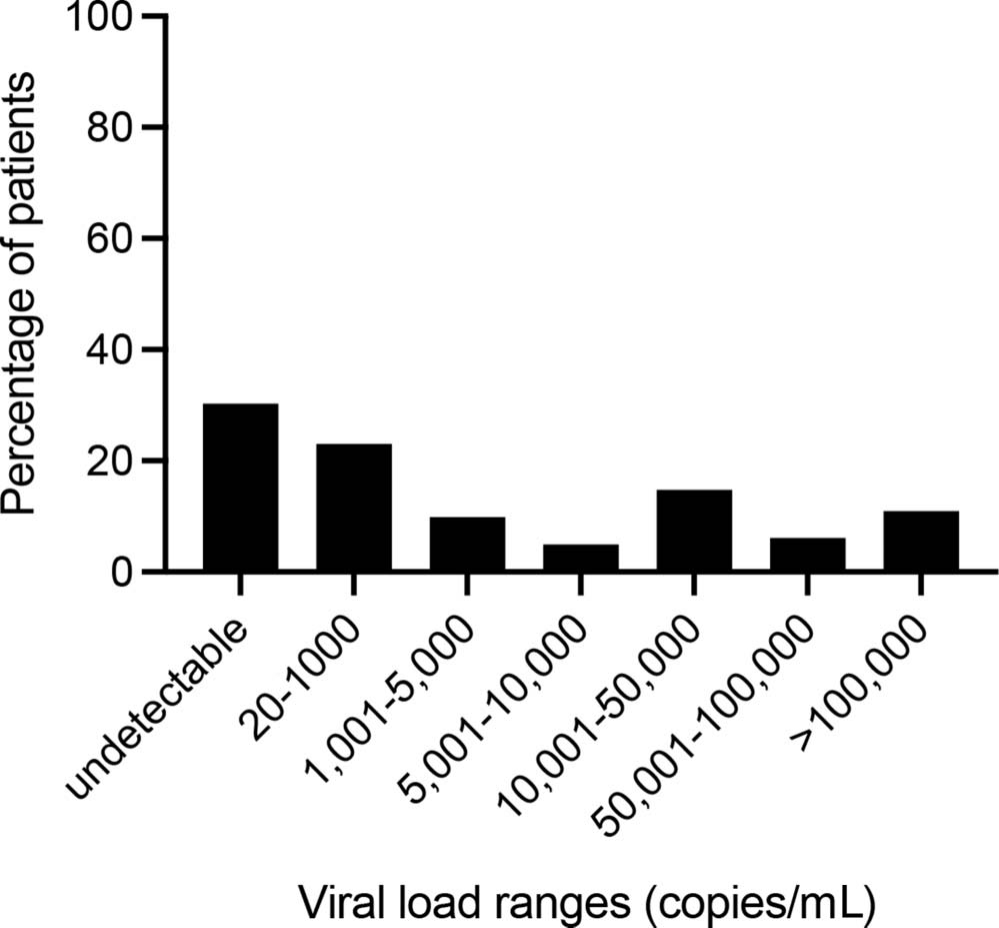
Patient plasma viral load distribution from all studies included in the meta-analysis.

Of the 23 studies included in the systematic review, 16 studies provided their primary data for a total of 18 data sets.^11,13–15,17–19,23,25–28,30–33^ Studies were reasonably distributed geographically with 53.0% of studies including study participants from Africa,^13,14,19,23,27,28,30,31,33^ 17.6% from Europe,^14,19,25^ and 29.4% from the United States/Canada/Mexico.^11,15,17,26,32^ This accounted for 1872 total paired dried plasma spot:plasma data points. The proportion of plasma values that was undetectable was 29.8%, whereas 70.2% was detectable (Fig. 3). A total of 23.1% of plasma values were between 20 and 1000 copies/mL, and 31.8% was greater than 10,000 copies/mL.

**FIGURE 4.**
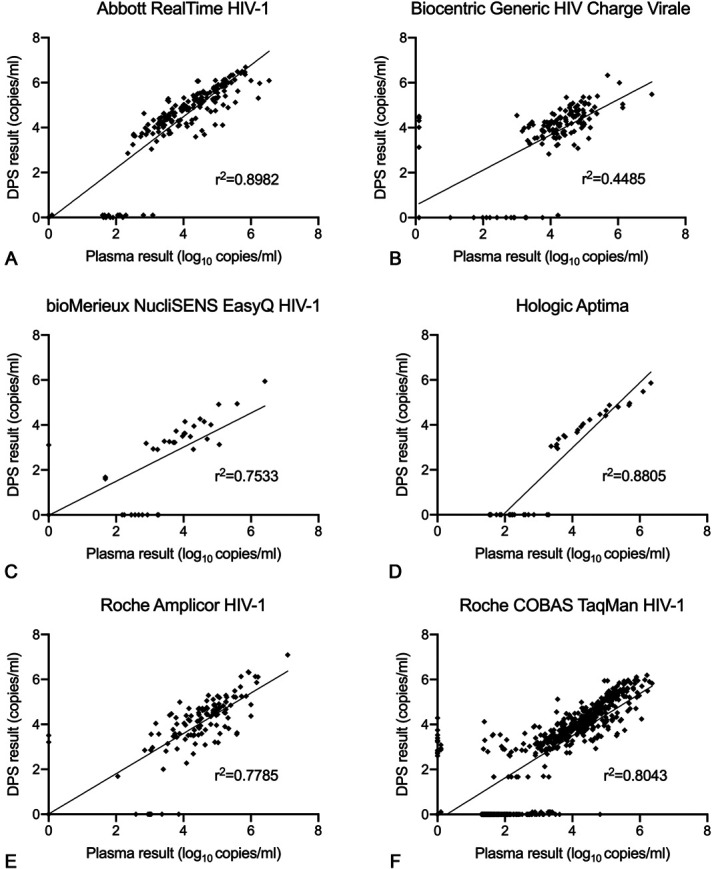
Meta-analysis linear regression graphs of each technology. A, Abbott RealTime HIV-1; (B) Biocentric Generic HIV Charge Virale; (C) bioMerieux NucliSENS EasyQ HIV-1; (D) Hologic Aptima; (E) Roche Amplicor HIV-1; (F) Roche COBAS TaqMan HIV-1.

The mean bias was 0.28 log_10_ copies/mL (dried plasma spot:plasma). All technologies had an r^2^ greater than 0.75, except for the Biocentric Generic HIV Charge Virale technology (r^2^ = 0.4485) (Fig. 4). For all technologies together, the median dried plasma spot viral load was 0.10 log_10_ copies/mL, whereas the median plasma viral load was 2.35 log_10_ copies/mL (Table 3). More dried plasma spot values were undetectable compared with plasma values (43.6% vs. 29.8%). There were a total of 560 undetectable plasma viral load results and 820 undetectable dried plasma spot results with 546 paired results being undetectable using both plasma and dried plasma spot. Ten of the 14 false detectable results using the dried plasma spot specimen were more than 1000 copies/mL with a median of 2250 copies/mL. There were 274 results that were detectable by plasma but undetectable by dried plasma spot specimen with a median plasma result of 56 copies/mL; however, only 20 had a plasma viral load result that was ≥1000 copies/mL. One hundred eighty of these 274 results (65.7%) had a plasma result that was less than 100 copies/mL.

**Table 3.** Meta-Analysis of the Clinical Metrics Overall and for Each Viral Load Technology

		All Technologies	Abbott RealTime HIV-1	Biocentric Generic HIV Charge Virale	bioMerieux NucliSENS EasyQ HIV-1	Hologic Aptima	Roche Amplicor HIV-1	Roche COBAS TaqMan HIV-1
	n	1847	245	148	183	40	154	1077
	Dried plasma spot:median viral load (log_10_ copies/mL)	0.10	4.42	4.12	0	2.99	4.01	0
	Plasma: median viral load (log_10_ copies/mL)	2.35	3.67	4.22	0	3.44	4.44	1.67
	Difference in medians (log_10_ copies/mL)	2.25	–0.75	0.1	0	0.45	0.43	1.67
	Mean bias (log_10_ copies/mL)	0.28	–0.36	0.3	0.18	1.27	0.38	0.39
	DBS:plasma threshold comparisons							
Sensitivity (UCL—LCL)	1000:1000	92.27 (87.58–95.28)	99.39 (95.78–99.91)	98.12 (56.78–99.95)	77.78 (53.53–91.40)	86.96 (66.45–95.73)	92.07 (78.12–97.42)	93.05 (87.75–96.16)
	800:800	93.72 (88.64–96.61)	99.40 (95.85–99.92)	98.13 (43.27–99.97)	84.21 (60.85–94.82)	91.30 (71.11–97.82)	90.42 (79.02–95.94)	93.91 (88.66–96.82)
	600:600	94.55 (90.01–97.10)	98.82 (95.42–99.71)	98.13 (44.03–99.97)	84.21 (60.85–94.82)	87.50 (67.62–95.91)	91.94 (85.66–95.61)	96.55 (89.36–98.94)
	500:500	94.39 (89.47–97.09)	98.84 (95.47–99.71)	98.13 (44.03–99.97)	80.00 (57.21–92.29)	84.00 (64.31–93.86)	91.94 (85.66–95.61)	96.85 (89.39–99.12)
	400:400	94.03 (88.97–96.85)	98.84 (95.47–99.71)	98.13 (33.64–99.98)	76.19 (53.97–89.73)	84.00 (64.31–93.86)	92.74 (86.64–96.18)	95.98 (88.40–98.68)
	200:200	93.67 (87.39–96.93)	98.87 (95.60–99.72)	98.14 (29.57–99.98)	66.67 (46.12–82.37)	77.78 (58.55–89.66)	92.00 (85.77–95.64)	95.58 (84.87–98.81)
	Detectable	97.06 (87.41–99.37)	97.83 (61.21–99.92)	—	88.53 (17.23–99.65)	—	93.65 (87.82–96.79)	99.79 (57.43–100.00)
Specificity (UCL–LCL)	1000:1000	95.57 (88.48–98.37)	85.37 (75.97–91.50)	75.00 (46.90–91.06)	99.39 (95.83–99.91)	100.00 (0.00–100.00)	95.56 (68.84–99.53)	94.90 (78.59–98.95)
	800:800	96.64 (89.79–98.95)	88.61 (79.53–93.96)	76.19 (1.90–99.81)	99.39 (95.80–99.91)	100.00 (0.00–100.00)	95.05 (67.97–99.43)	95.33 (78.11–99.15)
	600:600	97.11 (91.66–99.04)	90.67 (81.69–95.49)	74.99 (5.74–99.33)	99.39 (95.80–99.91)	100.00 (0.00–100.00)	93.33 (76.93–98.33)	98.05 (92.57–99.51)
	500:500	97.16 (91.80–99.05)	93.15 (84.58–97.12)	74.99 (5.74–99.33)	99.39 (95.78–99.91)	100.00 (0.00–100.00)	93.33 (76.93–98.33)	98.00 (92.97–99.45)
	400:400	96.51 (90.22–98.80)	93.15 (84.58–97.12)	72.22 (0.40–99.94)	99.38 (95.75–99.91)	100.00 (0.00–100.00)	93.33 (76.93–98.33)	97.24 (93.26–98.90)
	200:200	97.53 (92.46–99.22)	100.00 (0.00–100.00)	70.56 (0.19–99.97)	99.37 (95.67–99.91)	100.00 (0.00–100.00)	93.10 (76.25–98.27)	97.22 (93.59–98.82)
	Detectable	98.69 (95.03–99.66)	100.00 (0.00–100.00)	—	99.32 (23.05–100.00)	—	92.86 (75.52–98.21)	99.14 (92.67–99.90)
Total misclassification (UCL–LCL)	1000:1000	5.36 (3.26–8.69)	5.31 (3.11–8.92)	1.88 (0.02–64.28)	2.73 (1.14–6.39)	7.50 (2.44–20.82)	7.69 (3.23–17.24)	4.86 (3.28–7.13)
	800:800	4.73 (2.69–8.19)	4.08 (2.21–7.42)	1.87 (0.02–68.29)	2.19 (0.82–5.68)	5.00 (1.25–17.91)	9.08 (5.23–15.28)	3.94 (2.16–7.08)
	600:600	4.18 (2.23–7.70)	3.67 (1.92–6.91)	1.87 (0.02–68.28)	2.19 (0.82–5.68)	7.50 (2.44–20.82)	7.79 (4.48–13.22)	2.61 (0.86–7.65)
	500:500	4.31 (2.34–7.79)	2.86 (1.37–5.87)	1.87 (0.02–68.28)	2.73 (1.14–6.39)	10.00 (3.80–23.79)	7.79 (4.48–13.22)	2.58 (0.85–7.52)
	400:400	4.77 (2.80–8.02)	2.86 (1.37–5.87)	1.87 (0.01–74.96)	3.28 (1.48–7.10)	10.00 (3.80–23.79)	7.14 (4.00–12.44)	3.49 (1.49–7.95)
	200:200	4.76 (2.68–8.33)	0.82 (0.20–3.20)	1.87 (0.01–77.81)	4.92 (2.58–9.18)	15.00 (6.90–29.59)	7.79 (4.48–13.22)	4.02 (1.66–9.40)
	Detectable	2.81 (0.86–8.76)	1.86 (0.07–35.25)	—	6.01 (3.36–10.53)	—	6.49 (3.53–11.65)	0.60 (0.02–13.62)
Upward Misclassification (UCL–LCL)	1000:1000	4.43 (1.63–11.52)	14.63 (8.50–24.03)	25.00 (8.94–53.10)	0.61 (0.09–4.17)	0.00 (0.00–100.00)	4.44 (0.47–31.16)	5.10 (1.05–21.41)
	800:800	3.36 (1.05–10.21)	11.39 (6.04–20.47)	23.81 (0.19–98.10)	0.61 (0.09–4.20)	0.00 (0.00–100.00)	4.95 (0.57–32.03)	4.67 (0.85–21.89)
	600:600	2.89 (0.96–8.34)	9.33 (4.51–18.31)	25.01 (0.67–94.26)	0.61 (0.09–4.20)	0.00 (0.00–100.00)	6.67 (1.67–23.07)	1.95 (0.49–7.43)
	500:500	2.84 (0.95–8.20)	6.85 (2.88–15.42)	25.01 (0.67–94.26)	0.61 (0.09–4.22)	0.00 (0.00–100.00)	6.67 (1.67–23.07)	2.00 (0.55–7.03)
	400:400	3.49 (1.20–9.78)	6.85 (2.88–15.42)	27.78 (0.06–99.60)	0.62 (0.09–4.25)	0.00 (0.00–100.00)	6.67 (1.67–23.07)	2.76 (1.10–6.74)
	200:200	2.47 (0.78–7.54)	0.00 (0.00–100.00)	29.44 (0.03–99.81)	0.63 (0.09–4.33)	0.00 (0.00–100.00)	6.90 (1.73–23.75)	2.78 (1.18–6.41)
	Detectable	1.31 (0.34–4.97)	0.00 (0.00–100.00)	—	0.68 (0.00–76.95)	—	7.14 (1.79–24.48)	0.86 (0.10–7.33)
Downward misclassification (UCL–LCL)	1000:1000	7.73 (4.72–12.42)	0.61 (0.09–4.22)	1.88 (0.05–43.22)	22.22 (8.60–46.47)	13.04 (4.27–33.55)	7.93 (2.58–21.88)	6.95 (3.84–12.25)
	800:800	6.28 (3.39–11.36)	0.60 (0.08–4.15)	1.87 (0.03–56.73)	15.79 (5.18–39.15)	8.70 (2.18–28.89)	9.58 (4.06–20.98)	6.09 (3.18–11.34)
	600:600	5.45 (2.90–9.99)	1.18 (0.29–4.58)	1.87 (0.03–55.97)	15.79 (5.18–39.15)	12.50 (4.09–32.38)	8.06 (4.39–14.34)	3.45 (1.06–10.64)
	500:500	5.61 (2.91–10.53)	1.16 (0.29–4.53)	1.87 (0.03–55.97)	20.00 (7.71–42.79)	16.00 (6.14–35.69)	8.06 (4.39–14.34)	3.15 (0.88–10.61)
	400:400	5.97 (3.15–11.03)	1.16 (0.29–4.53)	1.87 (0.02–66.36)	23.81 (10.27–46.03)	16.00 (6.14–35.69)	7.26 (3.82–13.36)	4.02 (1.32–11.60)
	200:200	6.33 (3.07–12.61)	1.13 (0.28–4.40)	1.86 (0.02–70.43)	33.33 (17.63–53.88)	22.22 (10.34–41.45)	8.00 (4.36–14.23)	4.42 (1.19–15.13)
	Detectable	2.94 (0.63–12.59)	2.17 (0.08–38.79)	—	11.47 (0.35–82.77)	—	6.35 (3.21–12.18)	0.21 (0.00–42.57)
PPV (UCL–LCL)	1000:1000	96.21 (92.49–98.12)	93.10 (88.25–96.04)	98.12 (56.80–99.95)	93.33 (64.80–99.07)	100.00 (0.00–100.00)	98.87 (86.34–99.92)	95.39 (87.86–98.34)
	800:800	97.08 (93.51–98.72)	94.83 (90.36–97.29)	98.10 (66.38–99.93)	94.12 (67.97–99.18)	100.00 (0.00–100.00)	98.88 (85.95–99.92)	95.75 (87.35–98.66)
	600:600	98.03 (94.16–99.36)	96.00 (91.85–98.08)	98.10 (67.06–99.92)	94.12 (67.97–99.18)	100.00 (0.00–100.00)	98.70 (98.67–98.73)	97.69 (88.15–99.59)
	500:500	98.19 (94.50–99.42)	97.14 (93.32–98.81)	98.10 (67.06–99.92)	94.12 (67.97–99.18)	100.00 (0.00–100.00)	98.70 (98.67–98.73)	97.79 (88.27–99.62)
	400:400	97.77 (94.38–99.14)	97.14 (93.32–98.81)	98.10 (67.06–99.92)	94.12 (67.97–99.18)	100.00 (0.00–100.00)	98.37 (85.38–99.84)	96.74 (89.11–99.08)
	200:200	98.48 (95.55–99.49)	100.00 (0.00–100.00)	98.10 (67.06–99.92)	94.12 (67.97–99.18)	100.00 (0.00–100.00)	98.84 (86.15–99.91)	96.81 (89.60–99.07)
	Detectable	99.81 (96.59–99.99)	100.00 (0.00–100.00)	—	96.30 (77.92–99.48)	—	98.33 (93.58–99.58)	99.68 (82.86–99.99)
NPV (UCL–LCL)	1000:1000	88.28 (75.84–94.75)	98.59 (90.67–99.80)	75.00 (46.90–91.06)	97.62 (93.83–99.10)	85.00 (62.42–95.08)	74.57 (51.94–88.84)	93.67 (85.44–97.39)
	800:800	90.43 (76.37–96.51)	98.59 (90.67–99.80)	66.67 (37.52–86.95)	98.19 (94.55–99.42)	89.47 (66.26–97.35)	69.26 (49.13–84.02)	95.22 (85.77–98.50)
	600:600	90.85 (73.41–97.28)	97.14 (89.29–99.28)	65.22 (30.04–89.11)	98.19 (94.55–99.42)	84.21 (60.85–94.82)	72.49 (72.02–72.95)	95.96 (78.64–99.35)
	500:500	90.35 (73.18–96.98)	97.14 (89.29–99.28)	65.22 (30.04–89.11)	97.59 (93.76–99.09)	78.95 (55.45–91.87)	72.49 (72.02–72.95)	95.99 (79.82–99.31)
	400:400	89.67 (72.15–96.67)	97.14 (89.29–99.28)	56.52 (24.25–84.07)	96.99 (92.97–98.74)	78.95 (55.45–91.87)	75.48 (56.72–87.84)	95.20 (77.18–99.15)
	200:200	87.71 (68.53–95.90)	97.14 (89.29–99.28)	52.17 (25.77–77.41)	95.18 (90.66–97.57)	68.42 (45.16–85.08)	71.13 (50.46–85.63)	94.02 (67.87–99.15)
	Detectable	15.78 (0.43–89.06)	18.96 (0.05–99.04)	—	93.59 (88.50–96.52)	—	76.47 (59.54–87.77)	0.51 (0.00–99.85)

LCL, lower confidence limit; UCL, upper confidence limit.

At the treatment failure threshold of 1000 copies/mL, the sensitivity and specificity for all technologies together were 92.27% (95% CI: 87.58 to 95.28) and 95.57% (95% CI: 88.48 to 98.37), respectively (Table 3). The sensitivity and specificity of each technology were greater than 85% for nearly all technologies, with 2 exceptions: Biocentric Generic HIV Charge Virale (n = 148) had a high sensitivity (98.12%; 95% CI: 56.78% to 99.95%), but low specificity 75.00% (95% CI: 46.90% to 91.06%), and the bioMerieux NucliSENS EasyQ HIV-1 assay (n = 183) had a low sensitivity of 77.78% (95% CI: 53.53% to 91.40%), but a much high specificity of 99.39% (95% CI: 95.83% to 99.91%).

The sensitivity and specificity of dried plasma spot specimens at lower thresholds remained relatively consistent across all lower thresholds analyzed (Table 3). When considering a treatment failure threshold of any detectable result, the sensitivity and specificity were 97.06% (95% CI: 87.41 to 99.37) and 98.69% (95% CI: 95.03 to 99.66). The performance of dried plasma spot specimens across treatment failure thresholds also remained consistent compared with the 1000 copies/mL treatment failure threshold when analyzed for each technology. For all technologies together, the total, upward, and downward misclassifications were all less than 8% across each of the 7 treatment failure thresholds analyzed. All the technologies, with the exception of Biocentric Generic HIV Charge Virale (upward), bioMerieux NucliSENS EasyQ HIV-1 (downward), and Hologic Aptima (downward), had total, upward, and downward misclassifications of less than 15%.

## DISCUSSION

When dried plasma spot specimens were used for HIV-1 viral load testing, the diagnostic accuracy performance was relatively comparable with using traditional liquid plasma specimens. When analyzed across all technologies and treatment failure thresholds, the sensitivity and specificity remained greater than 92%. Furthermore, misclassification rates (total, upward, and downward) were low at less than 8%. These results are better and more consistent than a recent meta-analysis looking at the performance of dried blood spot specimens for viral load testing.^9^ This is most likely the case because the specimen type in the current meta-analysis was the same (plasma) as the comparator, whereas dried blood spot specimens consist of whole blood and are likely to detect intracellular RNA and proviral DNA and the standard, circulating RNA.^9,41^

Of interest, dried plasma spots were observed to sometimes have lower viral loads than the traditional liquid plasma specimens. In fact, 20.7% (274 of 1322) of all specimens that were detectable by plasma were undetectable by dried plasma spot specimens; however, only 20 of those plasma specimens (1.5% of 1322) were downward misclassified by the dried plasma spot specimen at the treatment failure threshold of 1000 copies/mL. The false undetectability observed was likely caused by the lower specimen input volume used for dried plasma spot specimens compared with the traditional liquid plasma. Most studies in this systematic review used 1–2 dried plasma spots of 50 mL each, yet 1 mm of plasma for the reference standard. Because of this, the limit of detection of dried plasma spots may be restricted by the smaller input volume and, thus, may not always detect those specimens with very low viral load values.

Although some challenges of false undetectability were observed, dried plasma spot specimens performed well and consistently at lower treatment failure thresholds. In fact, although the CIs were overlapping, the sensitivity and specificity were higher when a detectable treatment failure threshold was used compared with the 1000 copies/mL treatment failure threshold. This consistency should allow programs considering a lower treatment failure threshold to use this alternative specimen type if useful and feasible for their settings.

Programs across most high HIV burden countries still require novel solutions and innovations to improve access to viral load testing. Dried plasma spot specimens represent one potential innovation that may be able to support wider decentralization of viral load testing. One significant drawback to this technology, however, is the requirement for a centrifuge and human resource skills to separate plasma from the original whole blood specimen and spot onto the dried plasma cards. The spotting process, however, does not require traditional calibrated, scientific pipettes and techniques because each dried plasma spot takes a standard volume and the specimen can be applied using disposable plastic droppers or transfer dropper pipettes. The necessity for a centrifuge at the site of specimen collection is a significant challenge that may limit consideration. Furthermore, because most studies were conducted in developed settings, the feasibility in resource-limited settings is unclear, potentially limiting routine adoption. Alternative plasma separation methods would be helpful to allow for uptake and decentralization of this specimen type in settings in need of alternative approaches to access viral load testing.

Several alternative approaches have been developed more recently that try to take advantage of using plasma, yet with simplified preparation techniques that can be more accessible to resource-limited settings. Plasma separation or filtration devices or cards have been developed that allow for application of whole blood directly to the device or card that, with or without further manipulation, result in plasma that can then be used for viral load testing.^42–44^ Although these technologies may experience similar false undetectability challenges due to the small specimen input volume, the implications are likely to be similarly minor. Furthermore, such novel technologies will remove the requirement for on-site centrifugation and associated skills. However, as with any new specimen type, widespread uptake and decentralization require manufacturers to include alternative specimen types within intended use claims and regulatory submissions.

Most studies included in this systematic review analyzed their data with traditional quantitative measures, such as linear regression and Bland–Altman. Of interest, some studies did not include either metric, and there was poor consistency of the analyzed metrics across studies. Furthermore, no study analyzed their data considering the current application of viral load testing within the WHO recommendations and the treatment failure algorithm. This is likely primarily because most studies were conducted before the 2013 WHO guidelines when the WHO initially recommended viral load testing as the preferred modality to monitor treatment.^47^ A meta-analysis on this topic was, therefore, critical to provide a better understanding of the performance of dried plasma spot specimens for viral load testing. Furthermore, key metrics should be considered in all future diagnostic accuracy studies, using this or other sample types for viral load, including linear regression, Bland–Altman, and more qualitative metrics such as sensitivity/specificity and misclassification across a variety of treatment failure thresholds.

This study had several limitations. First, although the overall sample size of the meta-analysis was large and allowed for precise overall conclusions to be made, several technologies had relatively small sample sizes when each of the technologies were analyzed independently. More precise conclusions, therefore, could not be made for the Biocentric Generic HIV Charge Virale, Hologic Aptima, and Roche Amplicor HIV-1 technologies. Additional studies using these and upcoming technologies will allow for more meaningful interpretations. The Roche Amplicor HIV-1 technology has been discontinued and is no longer in use. Second, dried plasma spot preparation techniques varied across studies, particularly in the dried plasma spot card drying and storage time and conditions, the number of spots used, and the preparation protocol. Currently, none of the suppliers have validated dried plasma spots within their instructions for use or WHO prequalification documents; therefore, it is difficult to compare the protocols used in these studies with any standard protocol. Furthermore, there was not always consistency among the same technology. Fortunately, however, the results remained relatively consistent and CIs tight for those that had reasonable sample sizes. In addition, similar issues were observed in a recent meta-analysis reviewing the performance of dried blood spot specimens^9^; however, a subanalysis of manufacturer compliant studies did not perform significantly better. Third, although the studies spanned a number of countries and continents and could be considered generalizable, all studies conducted plasma separation and dried plasma spot specimen preparation in the laboratory from collected venipuncture specimens. This is unlikely to be the processing protocol if implemented in low-income and middle-income settings to support broader uptake of viral load testing; therefore, additional studies are necessary to understand the performance and feasibility of dried plasma spot specimens in intended use, more decentralized, health care facility settings. Fourth, the studies included in the meta-analysis had a considerable number of detectable specimens (nearly 70%), suggesting that the population included in these studies and/or meta-analysis may not be representative of current programmatic settings that typically have observed suppression rates of >80%.^45,46^ The positive and negative predictive values should, therefore, be cautiously interpreted. However, there were a substantial proportion of patients with low level viral loads (23% had a plasma viral load between 20 and 1000 copies/mL), and thus, the overall results remain informative. Fifth, unfortunately, not all authors shared primary data. Although nearly 70% of studies shared primary data for inclusion in the meta-analysis, the missing data could account for some potential bias in the results. Finally, due to the smaller sample sizes and lack of available patient demographic information, we were not able to conduct subanalyses focused on pediatric populations or people living with HIV who were on antiretroviral therapy.

This systematic review and meta-analysis provided strong evidence that dried plasma spot specimens can be used for accurate viral load testing. Manufacturers should consider incorporating this specimen type within official communications and regulatory submissions, whereas country programs and implementing organizations can consider the utility of this specimen type in an effort to further decentralize and expand access to viral load testing.
